# A minimal ligand binding pocket within a network of correlated mutations identified by multiple sequence and structural analysis of G protein coupled receptors

**DOI:** 10.1186/2046-1682-5-13

**Published:** 2012-06-29

**Authors:** Subhodeep Moitra, Kalyan C Tirupula, Judith Klein-Seetharaman, Christopher James Langmead

**Affiliations:** 1 Computer Science Department, Carnegie Mellon University, Gates Hillman Center, 5000 Forbes Avenue, Pittsburgh, PA, USA; 2 Department of Structural Biology, University of Pittsburgh School of Medicine, Rm. 2051, Biomedical Science Tower 3, 3501 Fifth Avenue, Pittsburgh, PA, USA

**Keywords:** GPCR, GREMLIN, Long-range interactions, Ligand binding pocket, Graphical model

## Abstract

**Background:**

G protein coupled receptors (GPCRs) are seven helical transmembrane proteins that function as signal transducers. They bind ligands in their extracellular and transmembrane regions and activate cognate G proteins at their intracellular surface at the other side of the membrane. The relay of allosteric communication between the ligand binding site and the distant G protein binding site is poorly understood. In this study, GREMLIN [1], a recently developed method that identifies networks of co-evolving residues from multiple sequence alignments, was used to identify those that may be involved in communicating the activation signal across the membrane. The GREMLIN-predicted long-range interactions between amino acids were analyzed with respect to the seven GPCR structures that have been crystallized at the time this study was undertaken.

**Results:**

GREMLIN significantly enriches the edges containing residues that are part of the ligand binding pocket, when compared to a control distribution of edges drawn from a random graph. An analysis of these edges reveals a minimal GPCR binding pocket containing four residues (T118^3.33^, M207^5.42^, Y268^6.51^ and A292^7.39^). Additionally, of the ten residues predicted to have the most long-range interactions (A117^3.32^, A272^6.55^, E113^3.28^, H211^5.46^, S186^EC2^, A292^7.39^, E122^3.37^, G90^2.57^, G114^3.29^ and M207^5.42^), nine are part of the ligand binding pocket.

**Conclusions:**

We demonstrate the use of GREMLIN to reveal a network of statistically correlated and functionally important residues in class A GPCRs. GREMLIN identified that ligand binding pocket residues are extensively correlated with distal residues. An analysis of the GREMLIN edges across multiple structures suggests that there may be a minimal binding pocket common to the seven known GPCRs. Further, the activation of rhodopsin involves these long-range interactions between extracellular and intracellular domain residues mediated by the retinal domain.

## Background

G-protein coupled receptors (GPCRs) are an important class of proteins initiating major biochemical pathways sensing environmental stimuli. They are the largest protein superfamily with an estimated 1000 genes in the human genome alone [2]. An estimated 30% of known drug compounds target these receptors [3]. Around 500 of GPCRs are odorant or taste receptors and the remaining bind endogenous ligands. The GPCR family is divided into five distinct classes, class A – E [4]. The class A family is the largest class and includes rhodopsin, the prototypical GPCR, for which the first crystal structure of any GPCR was solved [5]. Its ligand is 11-*cis* retinal (RT), covalently attached to the protein. *11-cis* RT isomerizes to *alltrans* RT upon light incidence, resulting in activation of the receptor. As of 2011, several additional GPCR structures have been deposited in the PDB increasing the total number of structures to 43 representing seven distinct GPCRs (Table 1). All GPCR structures are characterized by a transmembrane (TM) region consisting of seven helices, the G-protein interacting intracellular (IC) domain and an extracellular (EC) domain.

**Table 1:** GPCR summary table

**Receptor**	**PDB IDs [number of structures]**	**Ligands**
Bovine Rhodopsin (**BR**)	1 F88, 1GZM, 1HZX, 1JFP, 1L9H, 1LN6, 1U19, 2 G87, 2HPY, 2I35, 2I36, 2I37, 2J4Y, 2PED, 3C9L, 3C9M, 3CAP, 3DQB [18]	RT, Ligand free
Squid Rhodopsin (**SR**)	2Z73, 2ZIY [2]	RT
Turkey β1 adrenergic receptor (**β1AR**)	2VT4, 2Y00, 2Y01, 2Y02, 2Y03, 2Y04 [6]	Cyanopindilol, Dobutamine Carmoterol, Isoprenaline Salbutamol
Human β2 adrenergic receptor (**β2AR**)	2R4R, 2R4S, 2RH1, 3D4S, 3KJ6, 3NY8, 3NY9, 3NYA, 3P0G, 3PDS [10]	Carazalol, Timolol, ICI 118,551, (molecule from Kolb et al., 2009), Alprenolol, BI-167107, FAUC50
Human A2A adenosine receptor (**A2A**)	3EML [1]	ZM241385
Human chemokine receptor (**CXCR4**)	3ODU, 3OE0, 3OE6, 3OE8, 3OE9 [5]	IT1t, CVX15
Human dopamine D3 receptor (**D3R**)	3PBL [1]	Eticlopride

Summary of structural information available on GPCRs as of January 2011. While all ligands are small molecules, CVX15 (in bold) is a peptide.

In GPCRs, the binding of a ligand in the EC or TM domain is the signal that is propagated to the IC domain wherein different effectors bind, in particular the G protein heterotrimer, GPCR receptor kinases (GRK) and β-arrestin. Thus, receptor activation is an inherently allosteric process where the ligand binding signal is communicated to a distant site. The activation of rhodopsin and other class A GPCRs is thought to be conserved and involves rearrangements in structural microdomains [6]. Conformational changes of multiple ‘switches’ in tandem activate the receptor [7]. These long-range interactions between distant residues are important for the function of the receptors and are also closely involved in their folding and structural stability [8,9]. Identifying the residues involved in the propagation of signals within the protein is important to understand the mechanism of activation. While much information can be directly extracted from crystal structures, allosteric interactions are dynamic and implicit in nature and thus are not directly observable in static crystal structures. Experimental methods for investigating dynamics, such as nuclear magnetic resonance, are presently incapable of resolving allosteric interactions in large membrane proteins, such as GPCRs.

Due to the limitations of experimental methods, statistical analysis of GPCR sequences is an alternative in identifying residues that may be involved in allosteric communication. Here, considerable effort has been directed towards identifying networks of co-evolving residues from multiple sequence alignments (MSA), i.e. residues that are statistically correlated in the MSA. Such correlations are thought to be necessary for function, and may provide insights into how signals are propagated between different domains. A number of computational methods have been developed to identify such couplings from MSAs, including Hidden Markov Models (HMMs) [10], Statistical Coupling Analysis (SCA) [11,12], Explicit Likelihood of Subset Co-variation (ELSC) [13], Graphical Models for Residue Coupling (GMRC) [14], and Generative REgularized ModeLs of proteINs (GREMLIN) [1]. Like the GMRC method, GREMLIN learns an undirected probabilistic graphical model known as a Markov Random Field (MRF). Unlike HMMs, which are also graphical models, MRFs are well suited to modelling long-range couplings (i.e., between non-sequential residues). The SCA and ELSC methods return a set of residue couplings (which may include long-range couplings), but unlike MRFs, they do not distinguish between *direct* (conditionally dependent) and *indirect* (conditionally independent) correlations. This distinction is crucial in determining whether an observed correlation between two residues can be explained in terms of a network of correlations involving other residues. The key difference between the GMRC and GREMLIN methods is that GREMLIN is statistically consistent and guaranteed to learn an optimal MRF, whereas the GMRC uses heuristics to learn the MRF. We have previously reported detailed comparisons of the GMRC and GREMLIN methods [1] and found that GREMLIN achieved higher accuracy and superior scalability.

Multiple sequence alignments of class A GPCRs have previously been examined by the SCA [12] and GMRC [14] methods. In the SCA study, the authors focused on the critical residue at position 296 corresponding to a lysine (K296^7.43^), which is the covalent attachment site for RT in bovine rhodopsin [6,15]. Several networks of residues were proposed to mediate the signal flow from the ligand binding pocket to the G protein coupling site. This focus overlooked the important contribution of the EC domain to GPCR structure and dynamics [8]. In contrast to SCA, there were no statistically coupled residues involving K296^7.43^ in the GMRC study, rendering a comparison of SCA and GMRC results impossible. Only 5 edges in GMRC were considered statistically significant, limiting the interpretability of the results. At the time of the above studies, the rhodopsin crystal structure was the only GPCR structure available. The now larger number of structures published (Table 1) provides us with an opportunity to investigate the generality of the roles of individual residues for allostery in different GPCRs. Furthermore, we re-examine the communication across the entire membrane, not only from a single RT residue to the IC side, but considering all possible communication points.

Because of the demonstrated advantages of GREMLIN over other methods [1], we applied GREMLIN to the same GPCR sequence alignment previously investigated by SCA and GMRC studies for comparability [12,14]. Using GREMLIN we identified statistically significant long-range couplings in class A GPCRs and analyzed the results with respect to all seven GPCRs that had been crystallized at the time of our study. Our findings indicate that the ligand binding residues are significantly enriched in these long-range couplings, mediating not only communication to the IC, but also to the EC side of the membrane. 9 out of the 10 residues with the largest number of long-range couplings belong to the ligand binding domain. There a total of 34 statistically significant long-range couplings involving these 10 residues, involving experimentally determined microdomains and activation switches in GPCRs. Our study describes a comprehensive view of the network of statistical couplings across the membrane in class A GPCRs. The details of this network are consistent with the hypothesis that the ligand-binding pocket mediates allosteric communication. The independent identification of a crucial role of the ligand binding pocket in mediating this communication provides the first sequence-based support for the early notion that all three domains in GPCRs are structurally coupled [16]. Finally, the extent of enrichment of edges in different GPCR structures allowed us to propose a novel minimal binding pocket predicted to represent the common core of ligand contact residues crucial for activation of all class A GPCRs.

## Results and discussion

GREMLIN [1] was used to identify a network of correlated mutations in class A GPCRs. We first used bovine rhodopsin as a template to map the edges (correlations) to the structure. We defined the set of residues involved in interaction with the RT ligand based on the structure of rhodopsin, and first analysed the results with respect to these residues. Subsequently, we identified the ligand binding pockets of all GPCRs with known structure to consider generality of our findings. Finally, we identified minimal binding pockets that capture the most general aspects of ligand binding across all GPCRs we examined.

### Mapping of GREMLIN edges to the structure of bovine Rhodopsin

**Figure 1:**
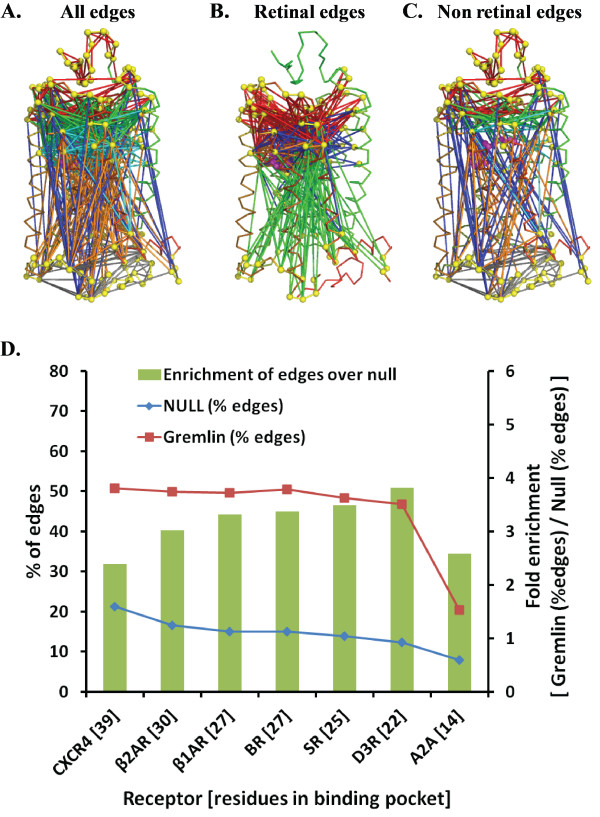
**Distribution of GREMLIN edges between different domains.** Mapping of (**A**) all (**B**) RT and (**C**) non RT edges identified by GREMLIN (at λ = 38) mapped onto the bovine rhodopsin structure (PDB ID: 1U19). The edges are EC-EC (red), EC-TM (green), EC-IC (blue), TM-TM (cyan), IC-TM (orange), IC-IC (grey40), EC-RT (red), RT-TM (blue), IC-RT (green) and RT-RT (orange) where EC, IC, TM and RT represent residues in extracellular, intracellular, transmembrane and RT (ligand binding) domains. In (**D**) The percentage of edges for GREMLIN (squares) and null set (diamonds) are plotted against the common ligand binding pockets sorted by their size. The bars indicate fold enrichment (values on secondary y-axis) of edges in GREMLIN over the null set

Our preliminary analysis (at regularization penalty λ = 38, see Methods) revealed that most edges involve residues in the RT ligand pocket, as compared to those between or within the residues belonging to EC, IC and TM domains outside of the RT pocket (Figure 1). The RT pocket is located in the TM domain, at the interface with the EC domain. To quantify the observation that there were differences in the number of edges connecting EC, IC, TM domains and RT pocket, we enumerated the GREMLIN edges and compared them to a control set, which included all possible edges (a total of 60,378 edges) involving all the 348 amino acids in rhodopsin. The results are summarized in Table 2. Assuming a significance level of α = 0.05, we find that there is a significant enrichment of edges involving RT residues compared to the control set (46.48% for GREMLIN vs. 13.87% for control; p-value of ~0). Similar enrichment was observed in the relative distributions of EC-EC (23.8% for GREMLIN vs. 6.78% for control; p-value of ~0) and IC-IC (14.93% vs. 7.24%, p-value ~0) edges. There was significant under-representation of edges in EC-IC (7.89% versus 14.17%, p-value ~ 0), EC-TM (21.55% versus 24.57%, p-value ~0.026) and IC-TM (11.41% versus 25.38%, p-value ~0). There was no significant difference in TM-TM contacts (20.42% versus 21.87%, p-value ~0.16).

**Table 2:** Comparison of edge distribution from control set and GREMLIN

**Categories**	**Control set (Null Distribution)**		**GREMLIN (at penaltyλ** **= 38)**		**GREMLIN > Null**	**GREMLIN < Null**
	**Total edges**	**% of edges**	**Total edges**	**% of edges**	**p value**	**p value**
**EC-EC**	4095	6.78	169	23.80	0	1
**EC-TM**	14833	24.57	153	21.55	0.97	0.03
**EC-IC**	8554	14.17	56	7.89	1	0
**TM-TM**	13203	21.87	145	20.42	0.84	0.16
**IC-TM**	15322	25.38	81	11.41	1	0
**IC-IC**	4371	7.24	106	14.93	0	1
**TOTAL**	**60378**	**100.00**	**710**	**100.00**		
**EC-RT**	2125	3.52	114	16.06	0	1
**RT-TM**	3600	5.96	98	13.80	0	1
**IC-RT**	2350	3.89	67	9.44	0	1
**RT-RT**	300	0.50	51	7.18	0	1
**SUB-TOTAL**	**8375**	**13.87**	**330**	**46.48**		

The finding that there is significant enrichment in the EC-EC and IC-IC contacts and that there is an under-representation of EC-IC domain contacts is biologically meaningful, because EC-IC interactions would structurally be mediated via the TM domain. Interestingly, there is a lack of significant enrichment of edges within the TM domain and a slight under-representation of EC-TM and TM-IC edges. A lack of TM enrichment is in line with the general view of the TM helices as rigid bodies in the GPCR field [17-19]. Furthermore, an important evolutionary pressure experienced by the amino acids in the TM region is to ensure that hydrophobic residues in the helices face the lipid bilayer. This pressure may override the importance of specific TM-TM contacts. However, it was puzzling that EC-TM and TM-IC contacts are under-represented since we would expect to find long-range couplings between EC and IC domains to be mediated via the intermediate TM domain. We therefore hypothesized that the EC-IC long-range contacts are more specifically mediated through a subset of TM and EC residues, namely those participating in binding RT. Indeed, 20 residues out of 27 in the RT pocket are in TM regions. We therefore analyzed the edges involving RT binding pocket residues in more detail.

### Long-range couplings involving the ligand binding pockets

The RT edges were further classified into EC-RT, RT-TM, IC-RT and RT-RT groups and were compared with the respective distributions in the control set. There is significant enrichment in EC-RT, IC-RT and all other groups compared to the control set (Table 2). This finding supports the hypothesis that the EC-IC long-range couplings are mediated via RT. This is in line with our current understanding of rhodopsin activation, as the initial conformational changes triggered on activation of the receptor are in the ligand binding domain which is ultimately propagated to the IC domain.

### Mapping of GREMLIN edges to the structure of other GPCRs

**Figure 2:**
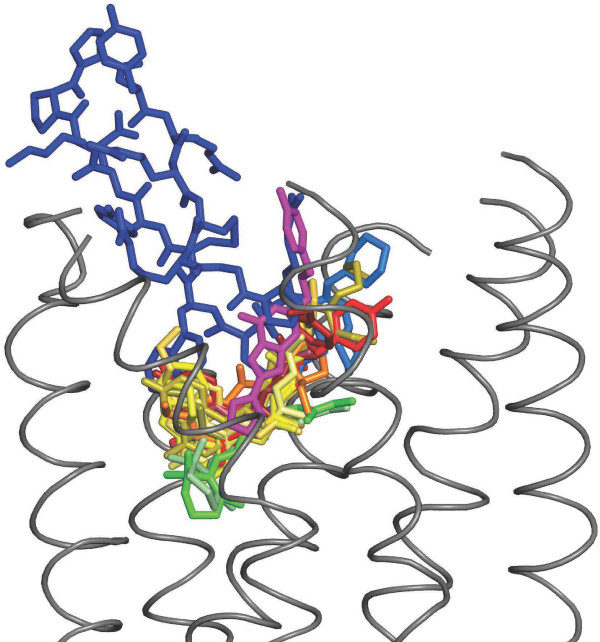
**Overlay of all ligands obtained from GPCR crystal structures mapped onto the structure of rhodopsin.** A total of 18 ligands from available GPCR structures are super positioned onto rhodopsin by structure alignment. The alignment and images were generated using PyMOL (Version 0.99rc6; http://pymol.org/pymol). For clarity loop regions and parts of TM regions of rhodopsin (in grey; PDB ID 1U19) are removed in the images. The ligands are colored as follows: BR (green), SR (pale green), β1AR (shades of red), β2AR (shades of yellow), A2A (magenta), D3R (cyan) and CXCR4 (CVX15 in blue and IT1t in marine blue). The PDB structures used in the figure are 1U19, 2Z73, 2VT4, 2Y00, 2Y02, 2Y03, 2Y04, 2RH1, 3D4S, 3NY8, 3NY9, 3NYA, 3P0G, 3PDS, 3EML, 3PBL, 3ODU and 3OE0. All the ligands show high overlap in their positions with RT, except CVX15, ZM241385 and IT1t, which only partially overlap with RT and other ligands

To extend this observation to other GPCRs, we defined a common binding pocket for each GPCR with known structure by taking the union of all residues in proximity to the ligands in cases where the same receptor was crystallized in the presence of multiple ligands (see Methods; Table 3). We compared the percentage of edges formed by the residues in these common binding pockets to that of the null distribution and against each other. As expected, the percentage of edges for the null set decreased linearly from 21% to 8% with decreasing number of residues in the pocket, i.e. pocket size (Figure 1). In contrast, the percentage of edges for the receptor binding pockets plateaus between 47% - 51%, independent of pocket size except for A2A, which had a lower value of 20% (Figure 1). The fold enrichment of edges for receptor binding pockets over the null set varied between 2.4 to 3.8. These results are statistically significant at significance level 0.05 with p-value ~ 0. Thus, GREMLIN significantly enriches edges containing ligand binding pocket residues compared to the control set. Importantly, the plateau observed in the percentage of edges for CXCR4, β2AR, β1AR, BR, SR and D3R suggests that there is a conserved ligand binding pocket shared between these receptors. The most probable explanation for the lower percentage of edges for A2A is that the ligand ZM241385 binds more towards the EC side compared to the position of ligands of other GPCRs (Figure 2). Additionally, it is oriented parallel to the TM helical bundle unlike ligands in other receptors in which a relatively perpendicular orientation is found. Nonetheless, the ligand binding pocket still contains an overlapping set of residue contacts with the other GPCRs (Figure 2). These findings suggest that there is a minimal binding pocket common to all GPCRs crystallized to date.

**Table 3:** Common ligand binding pockets defined for GPCRs with structural information

**CXCR4**	**β2AR**	**β1AR**	**BR**	**SR**	**D3R**	**A2A**
M1, G3, L31, Q36, F37, M44, T93, T94, T97, S98, F103, E113, G114, A117, T118, P171, L172, Y178, I179, P180, T193, P194, H195, E196, E197, N200, F203, V204, M207, Y268, A272, I275, H278, Q279, S281, P285, M288, T289, A292	M86, T94, T97, S98, F103, E113, G114, A117, T118, G121, E122, I179, P180, I189, Y191, F203, V204, M207, F208, H211, W265, Y268, A269, A272, P285, M288, T289, A292, F293, K296	T94, T97, S98, E113, G114, A117, T118, G121, E122, I179, P180, I189, Y191, F203, V204, M207, F208, H211, W265, Y268, A269, A272, M288, T289, A292, F293, K296	E113, G114, A117, T118, G121, E122, L125, Y178, E181, S186, C187, G188, I189, Y191, M207, F208, H211, F212, F261, W265, Y268, A269, A272, A292, F293, A295, K296	M86, G90, E113, G114, A117, T118, G121, E122, L125, Y178, E181, S186, C187, G188, I189, M207, F208, H211, F212, F261, W265, Y268, A269, A292, K296	T94, E113, G114, A117, T118, G121, E122, P180, G188, I189, V204, M207, F208, H211, W265, Y268, A269, A272, F273, M288, A292, K296	T118, P180, E181, F203, M207, W265, Y268, A269, A272, F283, P285, M288, T289, A292
**39**	**30**	**27**	**27**	**25**	**22**	**14**

The residues listed are analogous binding pockets mapped onto the rhodopsin structure (1U19). The binding pockets are arranged in the order of decreasing size of the binding pocket (left to right). The numbers in the last row represent the number of residues in the binding pocket.

### A minimal ligand binding pocket

**Figure 3:**
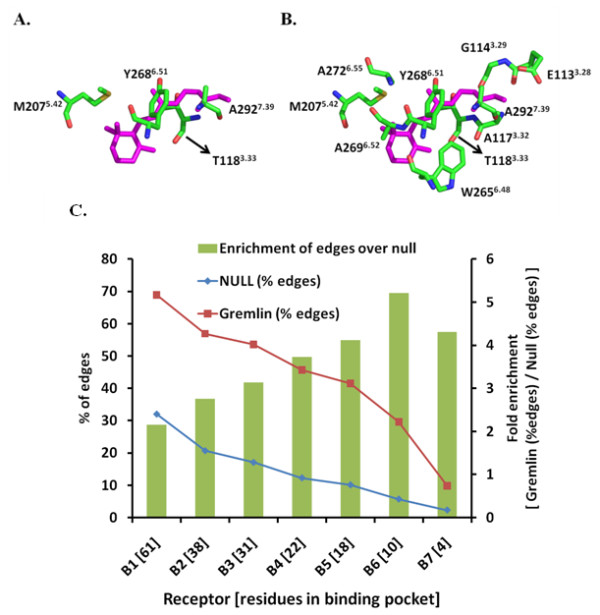
**Edge distributions in the minimal ligand binding pockets (GREMLIN vs. null set) and Location of minimal ligand binding pocket residues in rhodopsin structure.** The spatial organization of residues in the minimal binding pocket (**A**) B7 and the larger pocket (**B**) B6 as present in the rhodopsin structure (PDB id 1U19). Rhodopsin numbering along with Ballesteros-Weinstein numbering (superscript) is given for comparison with other GPCRs. For clarity only the binding pocket residues are shown along with bound RT (in magenta). The images were generated using PyMOL (Version 0.99rc6; http://pymol.org/pymol). In (**C**), the percentage of edges for GREMLIN (squares) and null set (diamonds) are plotted against the minimal ligand binding pockets sorted by their size. The bars indicate fold enrichment (values on secondary y-axis) of edges in GREMLIN over the null set

We hypothesized that if there is a minimal binding pocket common to the seven known GPCRs, then GREMLIN would significantly enrich the percentage edges for this pocket of residues compared to the null distribution set. To test this hypothesis we first defined ligand binding pockets B1, B2, B3, B4, B5, B6 and B7 representing residues common to at least one, two, three, four, five, six and seven receptor ligand binding pockets, respectively (Table 4). We compared the percentage of edges formed by the residues in these pockets to that of the null distribution set and against each other. The percentage of edges for the null set decreased linearly from 32% to 2% with decreasing pocket size (Figure 3). The percentage edges over the same range for GREMLIN decreased 69% to 10% as expected because of the decreasing pocket size. However, the fold enrichment of edges for GREMLIN over the null set increased from 2.2 – 5.2 for pockets B1 – B6. These results are statistically significant at a significance level of 0.05 with p-value ~ 0. The fold enrichment for B7 slightly decreased to 4.3 because the pocket is small with only 4 residues.

**Table 4:** Defining a minimal GPCR pocket

**B1**	**B2**	**B3**	**B4**	**B5**	**B6**	**B7**
M1, G3, L31, Q36, F37, M44, M86, G90, T93, T94, T97, S98, F103, E113, G114, A117, T118, G121, E122, L125, P171, L172, Y178, I179, P180, E181, S186, C187, G188, I189, Y191, T193, P194, H195, E196, E197, N200, F203, V204, M207, F208, H211, F212, F261, W265, Y268, A269, A272, F273, I275, H278, Q279, S281, F283, P285, M288, T289, A292, F293, A295, K296	M86, T94, T97, S98, F103, E113, G114, A117, T118, G121, E122, L125, Y178, I179, P180, E181, S186, C187, G188, I189, Y191, F203, V204, M207, F208, H211, F212, F261, W265, Y268, A269, A272, P285, M288, T289, A292, F293, K296	T94, T97, S98, E113, G114, A117, T118, G121, E122, Y178, I179, P180, E181, G188, I189, Y191, F203, V204, M207, F208, H211, W265, Y268, A269, A272, P285, M288, T289, A292, F293, K296	T94, E113, G114, A117, T118, G121, E122, P180, I189, F203, V204, M207, F208, H211, W265, Y268, A269, A272, M288, T289, A292, K296	E113, G114, A117, T118, G121, E122, P180, I189, M207, F208, H211, W265, Y268, A269, A272, M288, A292, K296	E113, G114, A117, T118, M207, W265, Y268, A269, A272, A292	T118, M207, Y268, A292
**61**	**38**	**31**	**22**	**18**	**10**	**4**

The residues listed are analogous binding pockets mapped onto the rhodopsin structure (1U19). The binding pockets are arranged in the order of decreasing size of the binding pocket (left to right). The numbers in the last row represent the number residues in the binding pocket. B1, B2, B3, B4, B5, B6 and B7 represent common sets of residues present in at least one, two, three, four, five, six and seven known receptor ligand binding pockets, respectively.

The four residues in B7 are T118^3.33^, M207^5.42^, Y268^6.51^ and A292^7.39^. These residues are uniquely positioned around the ligand (RT in rhodopsin; Figure 3) and make key interactions that stabilize RT [5,20]. On the other hand, the B6 pocket has the maximum enrichment of GREMLIN edges over the control set. There are 6 additional residues in B6 (for a total of 10 residues; E113^3.28^, G114^3.29^, A117^3.32^, T118^3.33^, M207^5.42^, W265^6.48^, Y268^6.51^, A269^6.52^, A272^6.55^, A292^7.39^) compared to B7 that seem to make contacts with RT towards the EC and IC side. These residues are known to stabilize ligand binding and are part of micro-domains that are involved in rhodopsin activation [5-7,20]. Thus, residues in B7 (T118^3.33^, M207^5.42^, Y268^6.51^ and A292^7.39^) form the minimal GPCR pocket but the expanded set of residues in B6 also represents a meaningful pocket for many GPCRs. Shown in Table 5 are all the edges formed by the residues in the minimal GPCR pocket, B7.

**Table 5:** **GREMLIN edges (**λ **= 38) involving residues from the B7 pocket**

**T118**	**M207**	**Y268**	**A292**
G90, T94, P171, E197, T198, H211, A269, A272, F293, M309, C316	G90, S98, G114, G121, E122, P171, E181, C185, D190, E196, A233, A269, I275, H278, G284, M288, T289, A292, F293, C316, K325, N326	NONE	A26, Y29, H65, L72, G90, T93, T94, V104, N111, A117, G121, N145, F148, S176, Y178, S186, D190, N199, N200, V204, M207, Q237, T243, A269, A272, I275, F276, Q312

Underlined residues form edges with at least two of the B7 pocket residues.

### Identification of the most frequently observed residues involved in long-range interactions in rhodopsin

The previous section showed that GREMLIN is able to shed light on the biological and structural properties of the GPCR family. In this section we present a strategy for ranking GREMLIN edges. This strategy can be used for exploratory purposes in order to discover novel couplings and residues that might play a key role in structure and function of the GPCR protein family.

The strategy is based on the following two key insights. The first insight is that the residues that have high degree in the graph of GREMLIN couplings could be considered as hubs that lie on the communication pathways in GPCRs. This is motivated by the graphical model since a mutation/perturbation in the hub residue could affect a number of other residues. The second insight is based on the persistence of certain couplings even under stringent model complexity constraints. The larger the regularization parameter, λ, the sparser the Markov Random Field (MRF), see Methods. Thus, each edge in the MRF can be assigned a persistence score equal to the maximum λ until which the coupling was retained. The persistence score is an indicator of the importance of the couplings and the corresponding residues.

We ranked the residues based on the number of edges at a penalty of λ=38. The number of edges shown in the set of top 10 residues most frequently involved in an edge is shown in Table 6. Nine of these top ten residues (A117^3.32^, A272^6.55^, E113^3.28^, H211^5.46^, S186^EC2^, A292^7.39^, E122^3.37^, G90^2.57^, G114^3.29^ and M207^5.42^) are part of the RT pocket and are involved in packing and stabilizing of RT [5,20]. Of these nine residues, eight are from the TM domain while S186^EC2^ is from the EC region. S186^EC2^ is involved in EC2 loop movement and its mutation to alanine alters the kinetics of activation [21,22]. The remaining residue G90^2.57^ that is not part of the RT pocket as defined by a 5 Å distance cut-off is nonetheless an important residue. The naturally occurring mutation G90^2.57^D in the RT degeneration disease, *Retinitis pigmentosa*, results in the constitutive activity of the receptor [23].

**Table 6:** List of top ranked residues and the most persistent edges

**Rank**	**Position**	**Number of edges (at λ = 38)**	**Most persistent pair position (edges at penalty λ = 140)**
1	**A117**^**3.32**^	41	G90^2.57^**,** E247 ^IC3^**, F293**^**7.40**^**, K296**^**7.43**^
2	**A272**^**6.55**^	30	L72^IC1^, **G114**^**3.29**^, S176^EC2^, Y178^EC2^
3	**E113**^**3.28**^	29	M44^1.39^, L72^IC1^, W126^3.41^, Q237^IC3^, **F293**^**7.40**^
4	**H211**^**5.46**^	29	F91^2.58^, C140 ^IC2^, F148 ^IC2^
5	**A292**^**7.39**^	28	Y29^EC (N-terminus)^
6	**S186**^**EC2**^	27	K67^IC1^, Q244^IC3^, P291^7.38^
7	**E122**^**3.37**^	26	I48^1.43^, G90^2.57^, E196^EC3^, **M207**^**5.42**^, **A269**^**6.52**^, **F293**^**7.40**^, C316^IC (C-terminus)^
8	G90^2.57^	23	**A117**^**3.32**^, G120^3.35^, **E122**^**3.37**^**, M207**^**5.42**^, Q237^IC3^, **A269**^**6.52**^, **F293**^**7.40**^
9	**G114**^**3.29**^	22	S176^EC2^, **A272**^**6.55**^, Y178^EC2^
10	**M207**^**5.42**^	22	G90^2.57^, **E122**^**3.37**^, C316^IC (C-terminus)^

Residues in bold are part of the RT binding pocket extracted from the rhodopsin structure (PDB ID: 1U19). The Ballesteros-Weinstein numbering (superscript) is given for comparison with other GPCRs. Only long-range edges are reported i.e. the edges formed with neighboring residues (8 amino acids on either side) are filtered out.

### Involvement of long-range interactions in activation of rhodopsin

**Figure 4:**
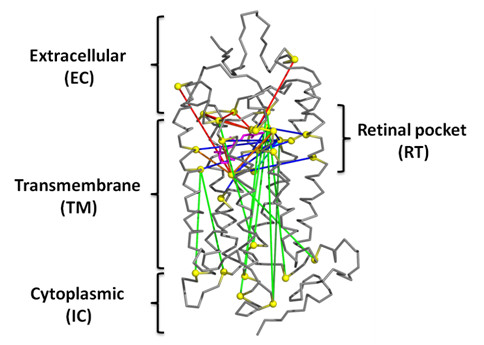
**Persistent long-range contacts mapped onto structure of rhodopsin.** Persistent edges at penalty 140 for the top 10 residues are mapped onto the rhodopsin structure (PDB id 1U19). The residues forming the edges are represented as yellow spheres. The edges are TM-TM (cyan), IC-TM (dark green), EC-RT (red), RT-TM (blue), IC-RT (green) and RT-RT (orange), where EC, IC, TM and RT represent residues in extracellular, intracellular, transmembrane and RT (ligand binding) domains, respectively. The image was generated using PyMOL (Version 0.99rc6; http://pymol.org/pymol)

The above analysis indicated that RT forms a central hub for long-range edges. This can also be seen intuitively from a plot of the edges at penalty 140 in the rhodopsin structure (Figure 4). For clarity, we discuss these residues in two groups, those involving the EC and TM domain and those involving the distant IC domain, separately.

### Edges involving the EC and TM domains

The RT attachment site, K296^7.43^, to which RT is covalently linked via a Schiff base with the amino group of this lysine, has 15 edges at λ = 38 and the most persistent edge is A117^3.32^ - K296^7.43^, the only long-range edge at λ = 280. K296^7.43^ is also a key determinant for ligand specificity in different GPCRs [6,15]. The counter-ion [24] for the Schiff base is E113^3.28^, also a top-ranked GREMLIN edge residue. The imine moiety of the RT Schiff base is surrounded by several amino acids of which M44^1.39^ and F293^7.40^ are identified in the edge list [19]. The major event on light-incidence is the isomerization of 11*-cis*-RT to all-*trans*-RT which results in the rotation of the C20 methyl group towards the EC2 loop [25]. This rotation triggers movements of the EC2 loop and rotation of the Schiff base to a more hydrophobic interior [7]. The EC2 loop displacement is one of the molecular switches in rhodopsin activation [7]. Three important residues that are part of this loop, namely S176^EC2^, Y178^EC2^ and S186^EC2^, are identified as top-ranked edges here.

Movement of EC2 is coupled to the outward rotation of the EC end of TM5. The shift in the RT β-ionone ring towards M207^5.42^ on TM5 results in a rearrangement of the hydrogen bonding network between this helix and TM3 [7]. Residue H211^5.46^ interacts with E122^3.37^ and W126^3.41^ and these interactions are important for receptor activation to form the Meta II state [26,27]. Other residues that are important for Meta II stability on TM3 and identified by GREMLIN are E113^3.28^, G114^3.29^, A117^3.32^, G120^3.35^, E122^3.37^ and W126^3.41^.

In addition to the rearrangement of the hydrogen bonding network between TM3 and TM5, RT isomerization in rhodopsin and ligand binding in GPCRs results in two major activation switches, the so-called rotamer toggle switch and the breakage of the ionic lock. Rotamer toggle switch refers to the rotation of W265^6.48^, a residue which is part of the conserved CWxP motif [28] causing reorientation of Y223^5.58^, M257^6.40^ and Y268^6.51^ on TM6 [7,29]. The conserved ionic lock involves the (E/D^3.49^)R^3.50^Y^3.51^ motif, Y223^5.58^ and E247^IC3^ at the IC side [30-32]. Note that R135^3.50^, Y223^5.58^ and W265^6.48^ did not appear in our edge lists because highly conserved residues naturally do not vary, and thus cannot co-vary, and so GREMLIN does not learn edges to/from such residues (see Methods). For the same reason, absent from our lists are residues from the highly conserved NPxxY motif [33] that are involved in the TM6 motions on the IC side. However, E247^IC3^ from the ionic lock which is not highly conserved is present in our list forming an edge with A117^3.32^. Other important residues that are present in our edge list are A269^6.52^, A272^6.55^ on TM6 and A292^7.39^ on TM7 which contribute to RT binding [5,20]. In addition, A269^6.52^ in rhodopsin is usually substituted by F^6.52^ in other GPCRs and is considered an extension of the conserved aromatic cluster on TM6. F^6.52^ is thought to act as ‘ligand-sensor’ in concert with the CWxP motif [34].

### Edges involving the IC domain

The IC domain is the domain that interacts with the G protein and other proteins of the signal transduction cascade and the communication of RT with this distant domain is thus of particular functional significance. Several IC residues (K67^IC1^, L72^IC1^, C140^IC2^, F148 ^IC2^, Q237^IC3^, Q244^IC3^, E247^IC3^, and C316^IC (C-terminus)^) form edges with the top ten residues that have the highest number of edges (Table 7). The conformational changes in the IC domain of rhodopsin that ensure receptor activation have been extensively investigated by cysteine mutagenesis coupled with biophysical studies of the cysteine mutants [35-39]. In rhodopsin, K67^IC1^C, F148^IC2^C, Q244^IC3^C display decreased G protein, in rhodospin called transducin (G_t_), activation compared to wild-type while L72^IC1^C has no effect on activation [38,40-42]. Moreover, solvent accessibility studies have shown that L72^IC1^C undergoes the largest conformational change in IC1 upon activation whereby it becomes more solvent exposed than in the dark state [37,41-43]. L72^IC1^ in the crystal structure of opsin makes Van der Waals contacts with the G_t_ peptide [30]. EPR studies show an increase in mobility of C140^IC2^, Q244^IC3^C on photoactivation while no such changes are seen for Q237^IC3^C and E247^IC3^C [40,43]. E247^IC3^ is a critical residue that forms a salt bridge with the conserved ionic lock motif and undergoes major conformational changes during activation leading to the formation of the G_t_ binding pocket [43]. C316 ^IC (C-terminus)^ is identified as a persistent edge and displays increased mobility upon activation by EPR studies [36,40].

**Table 7:** Persistent edges categorized based on the long-range contacts between different domains

**Edge category**	**Subset containing RT residues**
**EC – TM **[7]*	**EC – RT**[7]:
	A272^6.55^ - S176^EC2^, A272^6.55^ - Y178^EC2^, A292^7.39^ - Y29^EC (N-terminus)^, S186^EC2^ - P291^7.38^, E122^3.37^ - E196^EC3^, G114^3.29^ - S176^EC2^, G114^3.29^ - Y178^EC2^
**TM – TM **[17]	**TM(not RT) – TM(not RT) **[1]:
	G90^2.57^ - G120^3.35^
	**RT – TM **[10]:
	A117^3.32^ - G90^2.57^, E113^3.28^ - M44^1.39^, E113^3.28^ - W126^3.41^, H211^5.46^ - F91^2.58^, E122^3.37^ - I48^1.43^, E122^3.37^ - G90^2.57^, E122^3.37^ - M207^5.42^, G90^2.57^ - M207^5.42^, G90^2.57^ - A269^6.52^, G90^2.57^ - F293^7.40^
	**RT – RT **[6]:
	A117^3.32^ - F293^7.40^, A117^3.32^ - K296^7.43^, A272^6.55^ - G114^3.29^, E113^3.28^ - F293^7.40^, E122^3.37^ - A269^6.52^, E122^3.37^ - F293^7.40^
**TM – IC **[8]	**TM(not RT) – IC **[1]:
	G90^2.57^ - Q237^IC3^
	**RT – IC **[7]:
	A117^3.32^ - E247 ^IC3^, A272^6.55^ - L72^IC1^, E113^3.28^ - L72^IC1^, H211^5.46^ - C140 ^IC2^, H211^5.46^ - F148 ^IC2^, E122^3.37^ - C316^IC (C-terminus)^, M207^5.42^ - C316^IC (C-terminus)^
**EC – IC **[2]	**RT – IC **[2]:
	S186^EC2^ - K67^IC1^, S186^EC2^ - Q244^IC3^

*The number of edges in category is given in brackets.

Categorization of edges based on the long-range contacts between the EC, IC and TM domains. The number of edges are in each category are given in brackets. There are a total of 34 edges formed by top 10 ranking residues at penalty λ = 140. There are no edges in the EC – EC and IC – IC categories. In the second column, the edges are sub-categorized to include the RT domain. Ballesteros-Weinstein numbering (superscript) is given for comparison with other GPCRs. Only long-range edges are reported. These are edges where neighbouring residues (8 amino acids on either side) are filtered out. *The number of total edges is given in brackets.

### Involvement of long-range interaction residues identified by GREMLIN in ligand binding and function of angiotensin II type I receptor (AT1R)

To validate our findings using a GPCR not used in the present analysis and for which no structure is yet known, we chose the rat angiotensin II type I receptor (AT1R). AT1R is a class A GPCR which plays a vital role in cardiovascular physiology. Unlike rhodopsin, there is no full length structural or extensive biophysical data available for AT1R. However, pharmacological and structure-function properties of this receptor have been well studied by mutagenesis experiments [44].

Residues in AT1R that are homologous top ranking edge forming residues in rhodopsin were extracted based on the MSA used in GREMLIN analysis (Table 8; AT1R residues in the table and in the following text are highlighted by underlining to differentiate them from rhodopsin). Although rhodopsin and AT1R share only 20% sequence identity, general GPCR motifs such as the ionic lock and NPxxY on TM7 are conserved. In addition to these general features, we find that the subset of edges we discovered in this study have been independently shown by previous experiments to be important for ligand binding and the function of AT1R as discussed below.

**Table 8:** Residues in AT1R that are homologous top ranking edge forming residues in rhodopsin

**Ballesteros-Weinstein numbering**	**Rhodopsin residues**	**ATR1 residues**
**EC (N-Terminus)**	Y29	K20
**1.39**	M44	Y35
**1.43**	I48	F39
**IC1**	K67	M57
**IC1**	L72	A63
**2.57**	G90	L81
**2.58**	F91	P82
**3.28**	E113	A104
**3.29**	G114	S105
**3.32**	A117	V108
**3.35**	G120	N111
**3.37**	E122	Y113
**3.41**	W126	F117
**IC2**	C140	V131
**IC2**	F148	R140
**EC2**	S176	N168
**EC2**	Y178	F170
**EC2**	S186	V179
**EC2**	E196	S189
**5.42**	M207	K199
**5.46**	H211	G203
**IC3**	Q237	--
**IC3**	Q244	K232
**IC3**	E247	N235
**6.52**	A269	Q257
**6.55**	A272	T260
**7.38**	P291	T287
**7.39**	A292	I288
**7.40**	F293	C289
**7.43**	K296	Y292
**IC (C-Terminus)**	C316	Y312

Experimental and computational docking studies suggest that AT1R receptor agonist (angiotensin II [Ang II]) and antagonist (losartan) bind in the homologous RT binding site [44,45], thus hinting that many of the residues in the top ranking edge list may play a role in ligand binding in AT1R. Interestingly, the first step in AngII binding is thought to be the insertion of the C-terminus of the peptide in the receptor followed by the interaction of N-terminus residues of the peptide with EC and TM ends on the EC face [44]. AngII binding is supposed to extend from the EC face of the protein to the homologous RT binding site buried in the TM similar to peptide bound chemokine structure [46]. The carboxylate group on the C-terminus of AngII forms a salt bridge with K199^5.42^ on TM5 [47-49]. In addition, K199^5.42^ is also involved in insurmountable antagonism with carboxylate containing ligands [50]. Similar to K199^5.42^, Q257^6.52^ is also shown to be involved in insurmountable antagonism [51]. The C-terminal residue of AngII (F8) makes critical stacking interactions with the minimal binding pocket residue H256^6.51^ and the aromaticity of F8 and H256^6.51^ is important for receptor activation [49,52]. A N111^3.35^G mutation on TM3 results in constitutive activation of AT1R [53]. The mechanism of constitutive activation of the N111^3.35^G mutation is due its steric effects involving Y292^7.43^ on TM7 [54]. N111 is also required for discriminating AT1R specific ligands [55]. Other residues such as V179^EC2^ in the EC loop are also important for Ang II binding [56]. Residues like Y292^7.43^ in TM7, N235^IC3^ and Y312^IC(C-terminus)^ in the IC face are critical for G-protein coupling and second messenger generation in cells [57-59]. Thus, AT1R residues identified to be important by empirically performed mutagenesis experiments represent the bulk of the edges identified by GREMLIN, thus validating the applicability of our approach to other GPCRs, including those for which structural information is lacking.

### Comparison of results from GREMLIN with SCA and GMRC

Since we applied GREMLIN to the same MSA previously studied by the SCA [12] and GMRC [14] methods, we can directly compare, the residues found statistically coupled by the three methods, listed in Table 9. The GREMLIN residues correspond to those obtained at a penalty of λ = 38. In the SCA study, the authors focused on K296^7.43^, since this is a moderately conserved residue and a key determinant of ligand interaction in GPCRs [12]. The common residues between GREMLIN and SCA forming edges with K296^7.43^ are T93^2.60^, A117^3.32^, G121^3.36^ and F293^7.40^. There are no statistically coupled residues involving K296^7.43^ in the GMRC study (Table 9). There are only 5 edges in GMRC that are identified to be statistically significant and none of the residues that are identified have any edges in GREMLIN at a penalty of λ = 38. GMRC also shares no common edges with SCA. Only two out of five edges in the GMRC study qualify as long-range and the residues involved (A82^2.49^, C264^6.47^ and A299^7.46^) are strategically located in the middle of TM helices. This might be an artefact of the topology learning heuristic used by GMRC when compared with the other methods. It is important to note that in the GMRC study, the authors considered a sub-class of the original MSA [12] involving only amine (196 sequences), peptide (333 sequences) and rhodopsin (143 sequences) that represents the bulk of the sequences (672 out of a total of 948 sequences) [14].

**Table 9:** Comparison of edges reported in SCA and GMRC studies with GREMLIN

**GREMLIN**	**SCA **[12]	**GMRC **[14]
Residues involved in edges with K296 (at λ = 38)	Residues that are statistically coupled to K296 perturbation	Statistically coupled residues in amine + peptide + rhodopsin model
M44, L72, N73, G90, **T93**, G114, **A117**, **G121**, W175, Y178, C185, D190, S202, H211, A269, *P291, A292,****F293***	I54, T58, N73, N78, F91, T92, **T93**, E113, **A117**, **G121**, E122, I123, L125, V129, E134, Y136, F148, A164, F212, I213, I219, M257, F261, W265, Y268, ***F293****, F294, A295, S298, A299,****N302****,***F313**, M317	L57 – A82, *F313 – R314, I305 – Y306, N302 – I304,* C264 – A299
		**Note:** None of the above residues have any edges in GREMLIN (at λ =38)

Short range edges are italicized while bold residues are common edges between SCA and GREMLIN. Edges from GRMC are not shared by SCA or GREMLIN.

In the SCA study, the residues statistically coupled to K296^7.43^ were classified further into three classes: (1) Immediate neighbours - F293^7.40^, L294^7.41^, A295^7.42^, A299^7.46^, F91^2.56^, E113^3.28^, (2) Linked network - F261^6.44^, W265^6.48^, Y268^6.51^, F212^5.47^ and (3) Sparse but contiguous network: G121^3.36^, I123^3.38^, L125^3.40^, I219^5.54^, F261^6.44^, S298^7.45^, A299^7.46^, N302^7.49^. These categories were formulated on mapping the residues onto the rhodopsin structure. Residues in the immediate neighbour category are in the vicinity of K296^7.43^ and are mainly involved in helix packing interactions except for E113^3.28^. E113^3.28^ forms a salt bridge interaction with the protonated Schiff base on K296^7.43^ and is an important interaction identified by SCA. In the GREMLIN model, E113^3.28^ and K296^7.43^ aren’t connected by an edge, but they do share three common neighbours: M44, L72, and F293, and are thus indirectly correlated. The linked network residues in SCA are parallel to the membrane and form an aromatic cluster around the β-ionone ring of RT in rhodopsin. The residues in the sparse but contiguous network are distant from K296^7.43^ and form helix packing interactions toward the IC side. There are critical residues identified in the SCA study, most importantly W265^6.48^ which is part of the CWxP motif [28] and N302^7.49^ which is part of the NPxxY motif [33]. The SCA method performs a perturbation on a particular amino acid only if the corresponding sub-alignment size is beyond a certain cutoff in order to calculate ΔΔG_stat_ values. GREMLIN on the other hand makes no such distinction. Hence it is possible that SCA detects edges even if a position is fairly conserved whereas GREMLIN ignores them. This could be a source of difference between GREMLIN and SCA edge couplings. Overall, compared to SCA and GMRC, GREMLIN seems to identify couplings that are more extensive (i.e., involving EC, TM, RT and IC) and are part of experimentally functional switches and structural micro-domains that are critical for activation as discussed above.

### Limitations of the GREMLIN approach

GREMLIN is subject to the same kinds of limitations that all MSA-based analyses face. We briefly discuss these limitations here so that readers can better understand the nature of the results of our study.

GREMLIN is very sensitive to the size and contents of the MSA. A small, and/or poorly constructed MSA may result in subpar models. However, GREMLIN does attempt to deal with small MSAs (i.e., those with relatively few sequences) through regularization. As described in the Methods section, GREMLIN selects a value for the regularization parameter, λ, via a permutation of the columns of the MSA. Specifically, it selects a λ value that minimizes the expected number of false positive edges. It does this at the expense of an increase in the number of false negative edges. The value of λ is expected to be roughly inversely proportional to the number of sequences in the MSA. Likewise, the number of edges in the resulting model will be roughly inversely proportional to λ. So, small MSAs will inherently produce sparse graphs which will probably contain many “missing” edges that don’t have strong statistical support. Users must therefore consider the size of the MSA when interpreting the set of edges in the graph returned by GREMLIN.

In addition to the size of the MSA, the contents of the MSA are also important, especially if the MSA contains functionally heterogeneous sequences (as is the case in our study). In particular, weak signals in the MSA (e.g., due to sampling imbalances between different functional groups) are very likely to be missed. This is especially true for GREMLIN since it is biased towards minimizing false positive edges. Conversely, the GREMLIN algorithm will learn the conservation and correlation statistics for two (or more) divergent subclasses, provided that they are well represented in the MSA. Additionally, some of the edges learned by GREMLIN are due to correlations that distinguish functionally divergent sequences, while others are due to other constraints (e.g., conservation of charge). GREMLIN cannot distinguish between these two kinds of couplings. Naturally, one may attempt to compare the set of edges learned from functionally homogeneous MSAs to those learned from heterogeneous MSAs, but differences in the sizes of the MSAs can make it difficult to compare models, as discussed above. Addressing this limitation is one of our goals as part of on-going research.

As noted by an anonymous reviewer, GREMLIN is not well-suited to learning couplings between one residue and a cluster of functionally redundant residues (e.g., a cluster of Glu residues, any one of which could form a salt bridge with a nearby Lys), unless, the MSA contains examples of each possible clustering. Thus, care must be taken if the MSA contains such clusters.

Finally, the results presented in this paper are limited to GPCRs where the binding pocket is at or near the corresponding binding pocket of rhodopsin. Our MSA did not contain a significant number of GPCRs with binding pockets substantially different than rhodopsin, such as A2A.

## Conclusions

In this study we demonstrated the use of GREMLIN to identify a network of statistically correlated and functionally important residues in class A GPCRs. Based on sequence only, GREMLIN identified that ligand binding pocket residues are extensively correlated with distal residues, compared to those that are not part of the ligand pocket. An analysis of the GREMLIN edges across multiple structures suggests that there is a minimal binding pocket common to the seven known GPCRs. Statistically significant long-range couplings identified here were previously identified experimentally to be critical for activation of rhodopsin. Further, the activation of rhodopsin involves these long-range interactions between EC and IC residues mediated by RT. Compared to previously applied methods SCA and GMRC, GREMLIN identifies edges that span the entire protein and are functionally important. Based on our findings here with the GPCR family and our earlier studies with several soluble protein families [1], GREMLIN can be used to identify functionally important residue couplings in both soluble and membrane proteins. Future work can include validating the functional importance of novel residues and couplings identified by GREMLIN using molecular modelling tools such as GOBLIN [60] or via Molecular Dynamic Simulations and ultimately wet-lab experiments.

## Methods

### GREMLIN methodology

**Figure 5:**
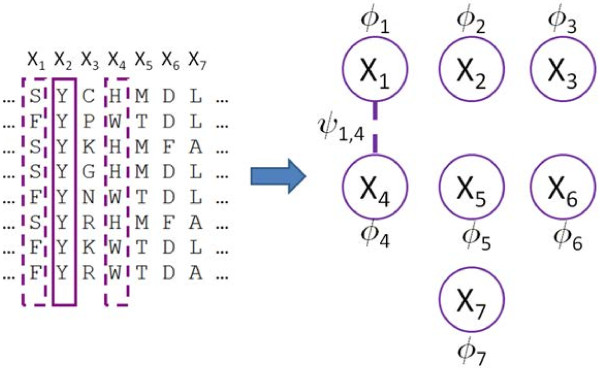
**Cartoon of a multiple sequence alignment and its mapping to a Markov random field.** Shown in the figure is a cartoon figure of a multiple sequence alignment (MSA) and a corresponding Markov random field (MRF). There is one node in the MRF for each column in the MSA. The column-wise conservation statistics in the MSA are encoded by node potentials (Φ_i_). Similarly, the co-variation statistics in the MSA (e.g., between columns 1 and 4) are encoded by edge potentials (φ_1,4_) in the MRF. The lack of an edge between two nodes means that the corresponding columns are conditionally independent.

We employed GREMLIN [1] to learn a Markov Random Field (MRF) model (Figure 5) from a MSA of class A GPCRs (see details below). MRFs are undirected probabilistic graphical models. In this paper, MRFs are used to model the conservation and coupling statistics observed in the MSA. In particular, each node in the MRF corresponds to a column in the MSA. An edge between two nodes indicates that they are coupled. Conversely, the absence of an edge between two nodes means that they are *conditionally* independent. The conservation and coupling statistics in a MRF are encoded via node (ϕ) and edge potentials (ψ). Informally, these potentials can be thought of as un-normalized probabilities. Collectively, these potentials encode the joint probability distribution over protein sequences such that the probability of any given length p sequence x = (x_1_, *x*_2_, …, x_p_) can be computed as:

(1) $$P_{M}(x)=\frac{1}{Z}\prod_{s\in V}\varphi_{s}(X_{s})\prod_{(s,t)\in E}\psi_{\mathrm{st}}(X_{s},X_{t})$$

Here, Z is the normalization constant, V and E are the nodes and edges in the MRF, respectively. We note MRFs are generative and can thus be used to sample new sequences (as in protein design).

Figure 5 shows a toy example of the relationship between the input MSA and the MRF that GREMLIN learns. Here, a 7-column MSA is shown. Column 2 is completely conserved, and is therefore statistically independent of the remaining columns. This independence is encoded in the MRF by the absence of an edge to the variable corresponding to the second column. On the other hand, columns 1 and 4 co-vary such that whenever there is an ‘S’ in column 1, there is a ‘H’ in column 4, and whenever there is an ‘F’ in column 1, there is a ‘W’ in column 4. This coupling is represented in the MRF by an edge between the variables corresponding to columns 1 and 4. In this paper, we examine the topology of the learned MRF to gain insights into the network of correlated mutations. Specifically, we are most interested in correlations that are observed between spatially distant residues from different domains of GPCRs.

### Multiple sequence alignment (MSA) of class A GPCRs

**Figure 6:**
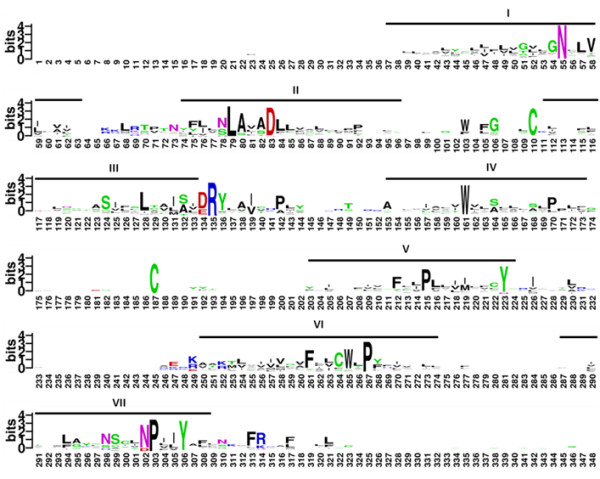
**Multiple sequence alignment of class A GPCRs.** For easy visualization the logo of the MSA alignment is generated using Weblogo ( http://weblogo.berkeley.edu). The amino acids numbering is based on the positions of bovine opsin (NCBI Reference Sequence: NP_001014890.1). The individual letter height of amino acid(s) at each position indicates their relative frequencies and conservation in the alignment. The TM helices are indicated as lines above the sequence. Most of the conserved regions are restricted to TM regions.

The authors of the SCA study [12] obtained the class A GPCR alignment from GPCRDB [61] and TinyGRAP [62] databases and manually adjusted the sequences using structure-based sequence alignments. The final MSA has 940 sequences and 348 residue positions covering the entire length of bovine rhodopsin without any gaps (Figure 6). We used this MSA here. As a pre-processing step, we selected the top 1000 candidate edges using a mutual information metric on which the structure learning approach would be subsequently run. This pre-processing step was done purely for computational reasons. Later versions of GREMLIN can avoid this pre-processing step by scaling up to larger sized proteins by parallelizing the computations using a Map-Reduce framework [63].

### Model selection

**Figure 7:**
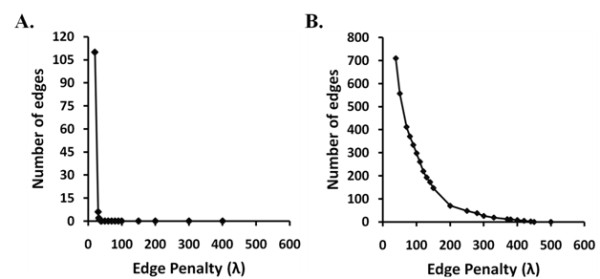
**Model Complexity Curve.** This figure shows a plot of the number of edges learned by the MRF as a function of the model complexity parameter, λ (**A**) on the permuted GPCR MSA (Null model) and (**B**) on the GPCR MSA. This exercise was carried out to define ‘robust’ edges or edges which are selected at a zero false positive rate. The penalty for which the number of edges goes to zero is λ = 38 for the permuted GPCR MSA and this is used as the parameter for defining ‘robust’ edges. The number of edges goes to zero at around λ = 450 for GPCR MSA. The smaller the parameter the denser the model and larger the parameter the sparser the model.

GREMLIN uses a single parameter, λ, which determines the sparsity of the MRF (i.e., the number of edges) and the likelihood of the sequences in the MSA under the model. Higher values of λ will produce sparser models. In general, a dense graph will yield higher likelihoods than a sparse graph. However, maximizing the likelihood of the MSA is likely to over-fit the data. Thus, the regularization parameter, λ, controls the trade-off between goodness-of-fit to the data and the tendency to over-fit. As in previous work, we used a permutation-based method to select λ. Briefly, we randomly permute the columns of the MSA in order to destroy all correlations between columns while retaining the column-wise distribution of amino acids. We then run GREMLIN on the permutated MSA using different values of λ. The smallest λ yielding zero edges on the permuted MSA is selected. This is a conservative estimate designed to minimize the number of false positive edges. A comparison of the number of edges versus λ for the permuted and the original alignment are shown in Figure 7A and 7B, respectively. In our experiments the optimal λ value was 38 (Figure 7A). We used GREMLIN to learn models from the un-permuted MSA using penalties of 38, or higher (Figure 7B). We consider such edges as the most “robust”. The analysis of GPCRs described here is based on these robust edges unless otherwise stated.

### GPCR structures files

As of January 2011, there were a total of 43 structures representing seven different GPCRs deposited in the PDB (Table 1). Only class A GPCRs have been crystallized so far. The GPCRs for which structural information is available are bovine rhodopsin (BR; 18 structures including opsin), squid rhodopsin (SR; 2 structures) turkey β_1_ adrenergic receptor (β1AR; 6 structures), human β_2_ adrenergic receptor (β2AR; 10 structures), human A_2A_ adenosine receptor (A2A; 1 structure), human chemokine receptor CXCR4 (5 structures) and human dopamine D3 receptor (D3R; 1 structure).

### Residue numbering scheme

The amino acids of the bovine rhodopsin sequence were used as position references (NCBI Reference Sequence [64]: NP_001014890.1). The positions of amino acids are represented by the single letter amino acid code followed by the sequence number in rhodopsin. To allow easier comparison with other GPCRs, given in superscript is the generic numbering proposed by Ballesteros and Weinstein [65].

### Description of ligand binding pockets in GPCR structures

The residues in the ligand pocket of the different GPCR crystal structures available to date were defined as those which have at least one atom within 5 Å of the respective ligand. Python scripts were written to extract residues within a ligand binding pocket using this cut-off distance from crystal structures.

We mapped the ligand binding pockets of the different GPCRs onto bovine rhodopsin for comparison. Pair-wise sequence/structure based alignments between rhodopsin (PDB ID: 1U19) and other GPCR structures were generated using the ‘salign’ module in the MODELLER [66] software. All ligand binding pockets discussed in this paper are mapped onto the structure of bovine rhodopsin.

In addition to comparing ligand binding pockets directly (i.e. extracting 5 Å residues in PDB ID: 1F88 for rhodopsin to identify the RT ligand binding pocket), we also generated the following combined sets of pocket residues to investigate similarities and differences between ligand binding pockets of different GPCRs (Table 1). For each of the 7 GPCRs, we defined a common ligand binding pocket by combining the ligand binding pockets from all available crystal structures for the respective receptor (Table 3). Thus, for bovine rhodopsin, the common ligand pocket is the combination of all RT binding pockets of 12 different structures. [Note: Rhodopsin PDBs excluded are 1JFP and 1LN6, because these represent structure models from NMR structures of protein fragments. 2I36, 2I37, 3CAP and 3DQB were also excluded because these are opsin structures and have no RT in them.] In analogous fashion, common pockets were created for squid rhodopsin (SR), turkey β_1_ adrenergic receptor (β_1_AR), human β_2_ adrenergic receptor (β_2_AR), human A_2A_ adenosine receptor (A2A), human chemokine receptor CXCR4 and human dopamine D3 receptor (D3R).

Finally, to generalize across different GPCRs, we derived additional ligand pockets B1, B2, B3, B4, B5, B6 and B7 representing common sets of residues present in at least one, two, three, four, five, six and seven receptor ligand binding pockets, respectively. These combined ligand binding pockets are listed in Table 4.

### Definition of long-range interactions

A long-range interaction is defined as a statistical coupling between two amino acids that are separated by at least 8 amino acids in the sequence (a definition used in CASP [13]).

### Control dataset and statistical significance tests

GREMLIN derived robust edges were checked for statistically over- or under-represented patterns amongst couplings observed. These tests were not done to validate the efficacy of GREMLIN in terms of modeling the protein family, but to get structural and biological insights into the nature of couplings that the model learns. For this purpose we compared the edges that GREMLIN returns against a control distribution of edges. The control distribution is created by drawing edges from a random graph. We classified the edges into one of the following categories: EC-EC, EC-IC, EC-TM, EC-RT, IC-IC, IC-RT, IC-TM, TM-TM, RT-TM and RT-RT. Here, RT stands for the ligand binding domain in rhodopsin (PDB ID: 1F88). To define the control distribution, we enumerated all possible edges coupling any two amino acids in rhodopsin (PDB ID: 1U19) and assigned these edges into the previously defined categories. We defined a control distribution of a category as the probability of randomly picking an edge in that category from the control dataset. To check for statistical significance, we enumerated the edges returned by GREMLIN in each category and compared the fraction of edges in this category against the control distribution. A p-value was calculated by a one-sided binomial test for statistical significance of GREMLIN categories against categories of the control distribution.

## Abbreviations

GREMLIN: Generative REgularized ModeLs of proteINs; GPCR: G protein coupled receptors; SCA: Statistical Coupling Analysis; GMRC: Graphical Models for Residue Coupling; HMM: Hidden Markov Model; MRF: Markov Random Field; MSA: Multiple sequence analysis; TM: Transmembrane; EC: Extracellular; IC: Intracellular; RT: Retinal; PDB: Protein data bank.

## Competing interests

The authors declare that they have no competing interests.

## Authors’ contributions

SM ran the GREMLIN experiments, participated in the design and performed the statistical analysis, and drafted the manuscript. KCT performed the structural analysis, participated in the design and statistical analysis of the study and drafted the manuscript. JKS and CJL conceived of the study, directed its design, coordinated all work and edited the manuscript. All authors read and approved the final manuscript.
